# Assessment of the pathogen genomics landscape highlights disparities and challenges for effective AMR Surveillance and outbreak response in the East African community

**DOI:** 10.1186/s12889-024-18990-0

**Published:** 2024-06-05

**Authors:** Julien A. Nguinkal, Yedomon A. B. Zoclanclounon, Andrea Molina, Abdi Roba, Ndia M. Nyakio, Peter N. Lokamar, Néhémie Nzoyikorera, Théogène Ihorimbere, Joseph Nyandwi, Mamdouh A. Aguer, James A. Maror, Michael Lasuba Lokore, Monica Fredrick Francis, Lawrence A. Mapunda, Medard Beyanga, Tonny Muyigi, Godfrey Pimundu, Susan N. Nabadda, Emmanuel Kabalisa, Jeanne d’Arc Umuringa, Isabelle Mukaga Tare, Hakim I. Lagu, Emmanuel Achol, Jürgen May, Muna Affara, Florian Gehre

**Affiliations:** 1 Department of Infectious Disease Epidemiology, Bernhard Nocht Institute for Tropical Medicine, Hamburg, Germany; 2 Plant Sciences and the Bioeconomy, Rothamsted Research, Harpenden, AL5 2JQ UK; 3 Department of Disease Surveillance and Epidemic Response, Ministry of Health, Nairobi, Kenya; 4 National Reference Laboratory, National Institute of Public Health, Bujumbura, Burundi; 5 National Public Health Laboratory, Ministry of Health, Juba, Republic of South Sudan; 6 National Public Health Laboratory, Ministry of Health, Dar es Salam, Tanzania; 7 Central Public Health Laboratories, National Health Laboratories, Ministry of Health, Kampala, Uganda; 8 Biomedical Services Department, Biomedical Centre Rwanda, Kigali, Rwanda; 9 Health Department, East African Community (EAC), Arusha, Tanzania; 10 Animal Science School, University of Costa Rica, San José, Costa Rica

**Keywords:** Public health surveillance, Outbreak response, Capacity-building, Pathogens data sharing, East African community, WHO priority pathogens, NGS capabilities, Pathogen genomics

## Abstract

The East African Community (EAC) grapples with many challenges in tackling infectious disease threats and antimicrobial resistance (AMR), underscoring the importance of regional and robust pathogen genomics capacities. However, a significant disparity exists among EAC Partner States in harnessing bacterial pathogen sequencing and data analysis capabilities for effective AMR surveillance and outbreak response. This study assesses the current landscape and challenges associated with pathogen next-generation sequencing (NGS) within EAC, explicitly focusing on World Health Organization (WHO) AMR-priority pathogens. The assessment adopts a comprehensive approach, integrating a questionnaire-based survey amongst National Public Health Laboratories (NPHLs) with an analysis of publicly available metadata on bacterial pathogens isolated in the EAC countries. In addition to the heavy reliance on third-party organizations for bacterial NGS, the findings reveal a significant disparity among EAC member States in leveraging bacterial pathogen sequencing and data analysis. Approximately 97% (*n* = 4,462) of publicly available high-quality bacterial genome assemblies of samples collected in the EAC were processed and analyzed by external organizations, mainly in Europe and North America. Tanzania led in-country sequencing efforts, followed by Kenya and Uganda. The other EAC countries had no publicly available samples or had all their samples sequenced and analyzed outside the region. Insufficient local NGS sequencing facilities, limited bioinformatics expertise, lack of adequate computing resources, and inadequate data-sharing mechanisms are among the most pressing challenges that hinder the EAC’s NPHLs from effectively leveraging pathogen genomics data. These insights emphasized the need to strengthen microbial pathogen sequencing and data analysis capabilities within the EAC to empower these laboratories to conduct pathogen sequencing and data analysis independently. Substantial investments in equipment, technology, and capacity-building initiatives are crucial for supporting regional preparedness against infectious disease outbreaks and mitigating the impact of AMR burden. In addition, collaborative efforts should be developed to narrow the gap, remedy regional imbalances, and harmonize NGS data standards. Supporting regional collaboration, strengthening in-country genomics capabilities, and investing in long-term training programs will ultimately improve pathogen data generation and foster a robust NGS-driven AMR surveillance and outbreak response in the EAC, thereby supporting global health initiatives.

**Supplementary Information:**

The online version contains supplementary material available at 10.1186/s12889-024-18990-0.

## Background

Bacterial pathogens cause infectious diseases that wreak havoc on Sub-Saharan African public health systems [1, 2]. Timely identification of these pathogens using next-generation sequencing (NGS) approaches is critical for effective disease surveillance, outbreak investigation, and the implementation of robust control measures [3–5]. Conventional methods, while valuable, frequently lack the resolution required for precise strain identification, transmission tracing, and understanding of the underlying genetic mechanisms, particularly in the context of emerging antimicrobial resistance (AMR) [5, 6]. The latest advancements in NGS technologies have paved the way for unprecedented pathogen surveillance, molecular epidemiology, and targeted public health interventions. Pathogen whole-genome sequencing (WGS) has been established as a powerful tool for unraveling the genomic landscape of microbial pathogens, providing intricate insights into their genetic makeup, evolution, and diversity [7–10]. This opens new avenues for detecting and responding quickly and effectively to disease threats and outbreaks [11–13].

The East African Community (EAC) is a regional intergovernmental organization that brings together eight East African countries, including Burundi, Kenya, Rwanda, Tanzania, Uganda, the Democratic Republic of the Congo (DRC), Somalia and South Sudan [14]. The EAC partner States, which have a combined population of approximately 285 million people, face a unique set of public health challenges related to infectious diseases. These challenges include high population density, inadequate or limited healthcare infrastructures, and various ecological and environmental factors that influence infectious disease dynamics [15, 16]. In this context, leveraging the full potential of pathogen genomics tools in public health is critical for effective disease management and control. As part of its commitment to timely and effectively addressing public health concerns in the region, the EAC is actively involved in several ongoing continental and global efforts to integrate and use genomics tools in public health. This includes strengthening National Public Health Laboratories (NPHLs) by facilitating the acquisition of state-of-the-art laboratory equipment, promoting training and capacity-building programs, and establishing a rapid response mobile laboratory network (RRMLN) equipped with molecular and NGS facilities [17–19].

Despite these promising efforts, substantial disparities persist among EAC Partner States in their capabilities to utilize NGS technologies to inform AMR monitoring and prevention strategies. These disparities are most evident in the limited ability to conduct in-country bacterial pathogen sequencing and bioinformatic analysis, particularly for the Global Antimicrobial Resistance and Surveillance System (GLASS) priority pathogens outlined in the World Health Organization’s (WHO) comprehensive global Genomic Surveillance strategy [20]. These pathogens include *Escherichia coli*, *Klebsiella pneumoniae*, *Staphylococcus aureus, Streptococcus pneumoniae*, *Salmonella spp.*, *Acinetobacter spp.*, *Shigella spp.*, and *Neisseria gonorrhoeae* [20]. National, regional, and global surveillance reports highlight the alarming prevalence of AMR in pathogenic bacteria within African countries, including the EAC Partner States [21, 22]. This poses a significant threat to public health, necessitating the adoption of modern AMR surveillance tools like pathogen NGS. South Africa, for example, stands out as a pioneer on the continent with a well-developed pathogen genomics infrastructure. Their successful application of these capabilities to improve public health surveillance and preparedness serves as a model for other African countries [23]. In contrast, EAC member States face diverse constraints in establishing, operating and sustaining in-country pathogen NGS capacities.

This study assesses the pathogen genome sequencing capabilities of the EAC by analyzing genome assembly metadata from bacterial isolates, focusing mainly on GLASS AMR-priority pathogens. Our objectives are two-fold: first, to evaluate the current sequencing capabilities within the EAC and identify challenges these countries encounter in integrating NGS pathogen data into public health systems; second, to benchmark these capabilities against South Africa’s established pathogen NGS infrastructure. We document the region’s pathogen NGS output and efforts by analyzing sequence metadata such as the isolation countries, sequencing platforms used, and the roles of submitting organizations or laboratories. Insights from a questionnaire-based survey helped pinpoint existing gaps and challenges while identifying opportunities for enhancement and further development. This study underscores the urgent need for substantial investments and commitments from both governmental and international partners to bolster the EAC’s bioinformatics and pathogen genomic capabilities. We also provide specific recommendations to narrow these gaps and reduce disparities between EAC Partner States and advanced Northern countries, emphasizing the importance of establishing sustainable, local and in-country capacities.

## Bacterial NGS data assessment in EAC

### Data collection and wrangling

Data on bacterial genome assemblies were obtained from international sequence repositories, including GenBank, BioSamples, and the Bacterial and Viral Bioinformatics Resource Center (BVBRC). The repositories were queried on different days due to the time required for retrieving and downloading metadata and genome sequences. Access occurred between March 30, 2023, and April 10, 2023. These databases provide pathogen sequence data with contextual information about samples and experimental settings. We investigated the metadata attributes of isolation countries, sequencing platforms, genome quality, submission date, and the role of submitting organizations or laboratories.

The data retrieved from various sources was integrated while merging disparate records to eliminate redundancy and streamline the dataset into a coherent format. We then performed preprocessing of the data, including cleaning and filtering, to ensure the integrity of the relevant variables. Preprocessing metadata involved removing inconsistent entries between databases and discarding records with missing information, such as isolation countries, sequencing centers, and data owners. Only plasmid and genome sequences flagged as “poor” quality were removed from genomic data. These steps were necessary to ensure the reliability and consistency of downstream analyses. We limited our study only to bacterial isolates from the EAC countries. Finally, we limited the temporal scope to the last 15 years, dating back to 2008. This temporal constraint ensured our analysis remained contemporary and reflected recent advances in NGS technologies.

We conducted an online questionnaire-based survey between October 30 and November 14, 2023, to assess the NGS capabilities and challenges in six NPHLs in the EAC. These NPHLs included Burundi, Rwanda, Tanzania, Kenya, Uganda, and South Sudan. The questionnaire, which included closed-ended and open-ended questions, aimed to collect quantitative data and qualitative insights into various aspects of pathogen NGS capabilities and implementation. These aspects included infrastructure, expertise, workflows, challenges, and perspectives or expectations for pathogen NGS implementation in their laboratories. The full questionnaire and raw responses are available in Supplement Files 1 and 2.

### Data analysis

**Fig. 1:**
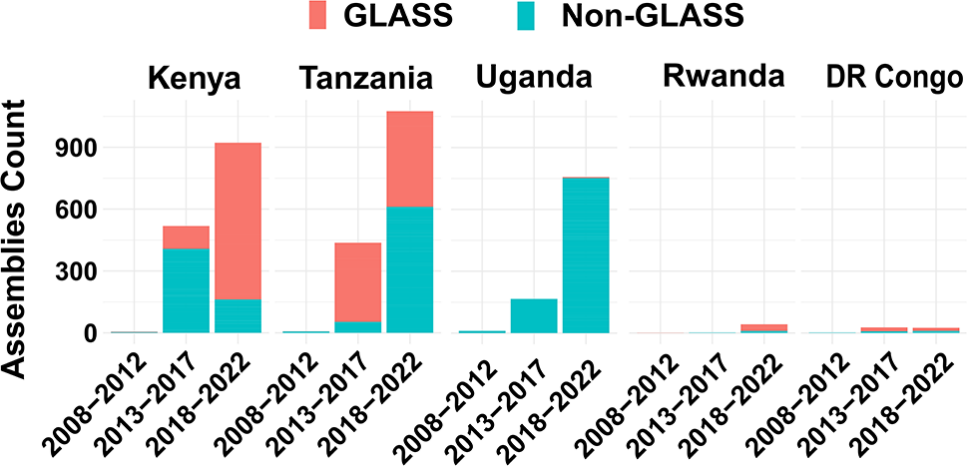
Temporal trends in the evolution of publicly available bacterial sequences, including AMR-GLASS priority pathogens and other non-GLASS bacteria, for each EAC partner state over the last 15 years, beginning in 2008. Only countries with at least five sequences are displayed for clarity. Burundi and South Sudan are not depicted, as Burundi has two publicly available sequences of non-GLASS pathogens, and South Sudan currently has no publicly accessible pathogen sequences data

**Fig. 2:**
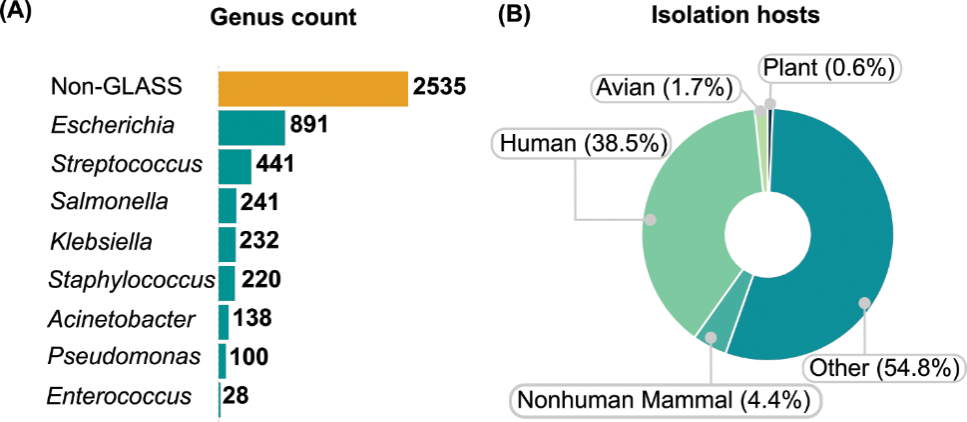
Summary bacterial genome sequences by species and isolation hosts. Cumulative count of bacterial genome sequences at the genus level, including GLASS and non-GLASS pathogens (**A**); Isolation hosts of GLASS-Priority pathogens (**B**)

Over the past five years, the number of publicly accessible bacterial isolate sequences from the EAC region has substantially increased. Starting with only 700 datasets in 2018, the number of complete bacterial genome sequences has risen to over 4,600 high-quality bacterial sequences by 2022 (Fig. 1). Tanzania leads in terms of overall bacterial genome sequences (*n* = 3,267), with 68% (*n* = 2,223) representing GLASS pathogens proportion. Kenya follows, with 2,863 sequences, of which 50.4% (*n* = 1,443) are AMR-GLASS pathogens. Despite a lower sequence count (*n* = 145), Rwanda has a GLASS sequencing rate of 31% (*n* = 45). In contrast, Burundi reported only two bacterial genomes, whereas South Sudan lacked publicly available data. Approximately 43.5% of all EAC-isolated samples were GLASS-priority pathogens, and 38.5% of these pathogens had humans as isolation hosts. These pathogens were predominantly *Escherichia coli, Streptococcus spp.*, and *Salmonella spp*., with 891, 441, and 232 sequences, respectively (Fig. 2). When considering in-country sequenced GLASS-priority pathogen samples, Tanzania (*n* = 134; 6%) led, followed by Kenya (*n* = 44; 3%) and Uganda (*n* = 4; 0.5%). When assessing unsubmitted data in the NPHLs, Kenya had the most bacterial sequences (*n* = 546), followed by Uganda (*n* = 85) and Tanzania (*n* = 10), while the other countries reported no unsubmitted sequences (Table 1). We also noticed that no EAC countries had sequence data on *Shigella species* and *Neisseria gonorrhoea*, which is concerning as these species are among the high-priority GLASS-AMR pathogens monitored by the WHO.

**Table 1:** Summary of total number of AMR-GLASS pathogens sampled in processed by EAC’s NPHLs between 2018 and 2023

Species	Kenya	Burundi	Rwanda	Uganda	Tanzania	South Sudan	DR Congo
*Acinetobacter spp.*	13	0	0	0	0	0	0
*Escherichia coli*	346	0	0	0	0	0	0
*Klebsiella pneumoniae*	34	0	0	0	10	0	0
*Neisseria gonorrhoeae*	0	0	0	0	0	0	0
*Salmonella spp.*	153	0	0	65	0	0	0
*Shigella spp.*	0	0	0	0	0	0	0
*Staphylococcus aureus*	0	0	0	0	0	0	0
*Streptococcus pneumoniae*	0	0	0	0	0	0	0

### Antimicrobial profile analysis

**Fig. 3:**
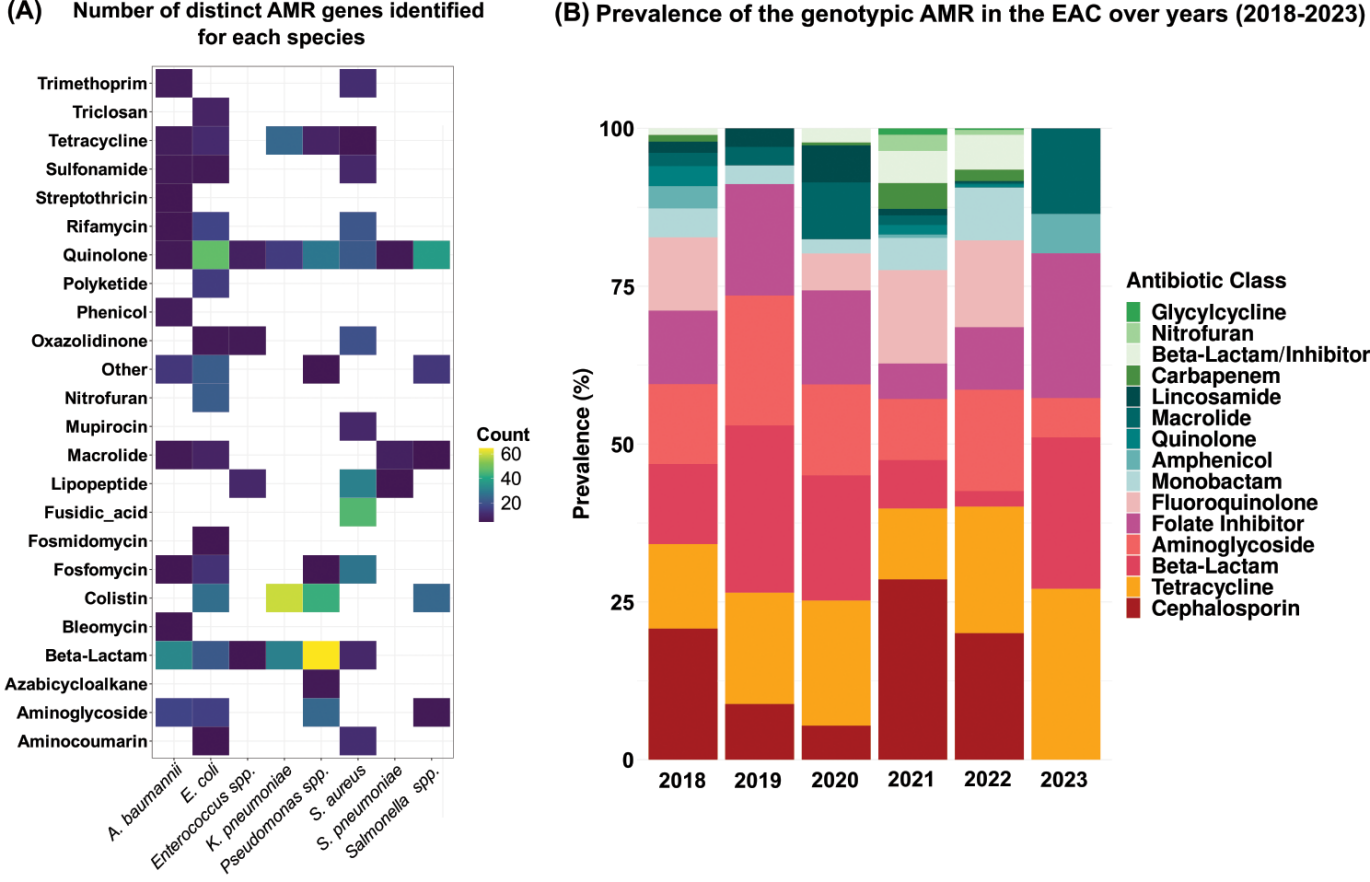
Antibiotic resistance profiles and key AMR genes in bacterial isolates in the EAC region. (**A**) summarizes the prevalence of predicted resistance genes. This heatmap shows the number of resistance genes identified for different bacterial species and antibiotic classes in the East African Community (EAC) region. Dark purple color indicates a lower number of genes identified. White boxes indicate no resistance was predicted for that specific antibiotic class. (**B**) shows temporal trends in antibiotics resistance patterns to different antibiotic classes. The stacked bar graph depicts the overall prevalence of predicted genotypic resistance profiles in the EAC region for each year between 2018 and 2023

**Fig. 4:**
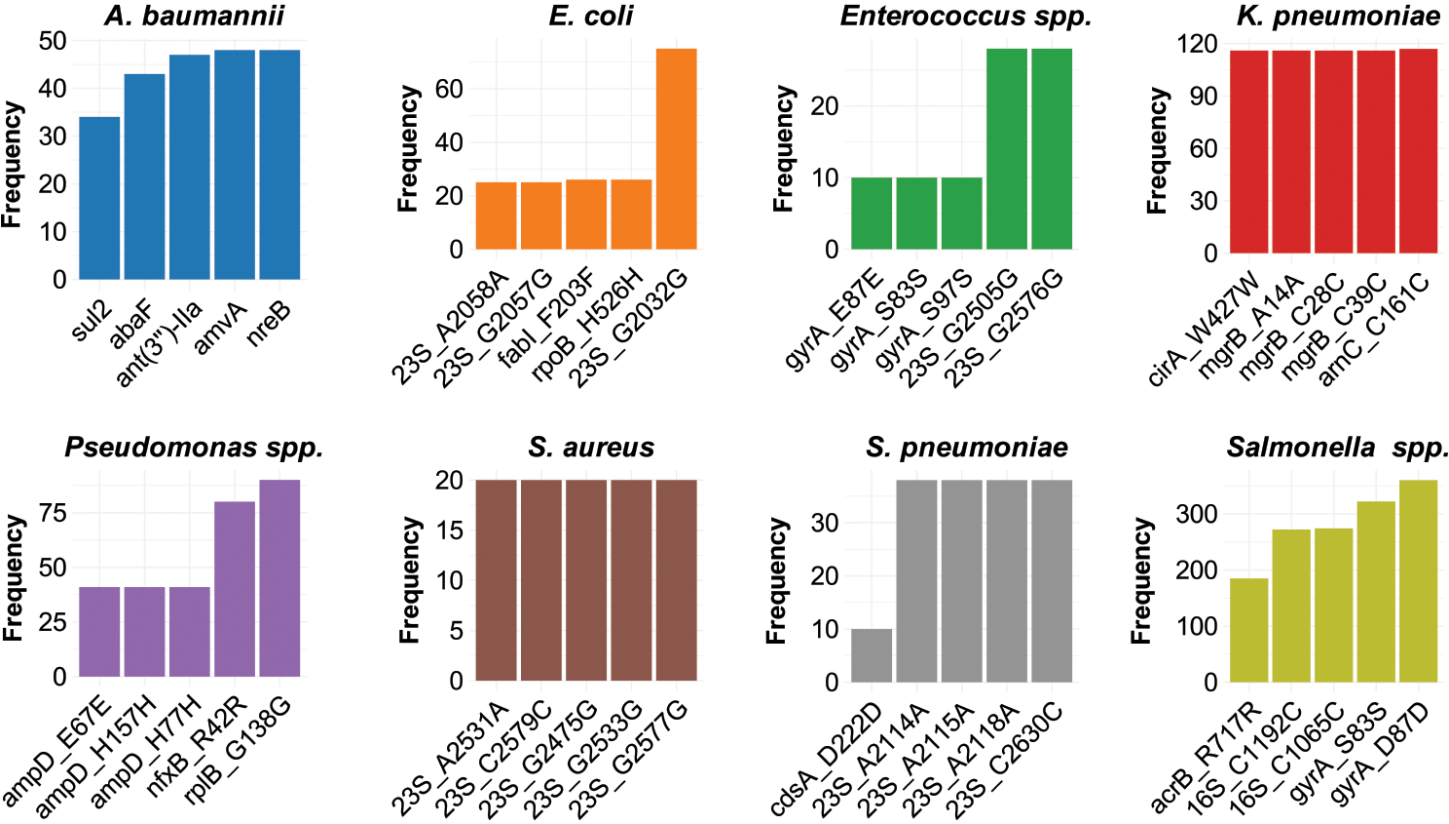
Most prevalent antibiotic resistance genes identified in bacteria isolates in the EAC region. The bar graphs depict the frequency of the top 5 predicted antibiotic resistance genes (ARGs) identified for each bacterial species isolated in the East African region. Known ARGs were predicted on whole genome sequences using both AMRFinder and STARAMR pipelines

To identify the AMR determinants associated with these bacterial sequences, we utilized two well-known tools, AMRFinder [23] and StarAMR [24], for predicting genotypic AMR profiles in the bacterial dataset. These tools analyze genomic data to identify known AMR genes (ARGs) and predict resistance phenotypes to specific antimicrobial agents. Additionally, we conducted a longitudinal analysis to examine the prevalence of resistance determinants in the EAC region over the past five years. The data indicate that more than 75% of isolates exhibited resistance to at least one drug from the classes of cephalosporin, tetracycline, beta-lactam, aminoglycoside, folate inhibitors, and fluoroquinolone (Fig. 3). Several ARGs demonstrate high prevalence across multiple bacterial species. Notably, the *gyrA_D87D* gene shows a substantial frequency among *Salmonella* isolates. However, certain genes appear to be specific to particular bacterial species. For instance, the *arnC_C161C* gene was predominantly found in *K. pneumoniae*, while the resistance gene *23S_G2032G* was more prevalent in *E. coli* (Fig. 4, Supplement file 3).

## Challenges and opportunities of pathogens genomics

### Current challenges in NGS pathogen analysis

**Fig. 5:**
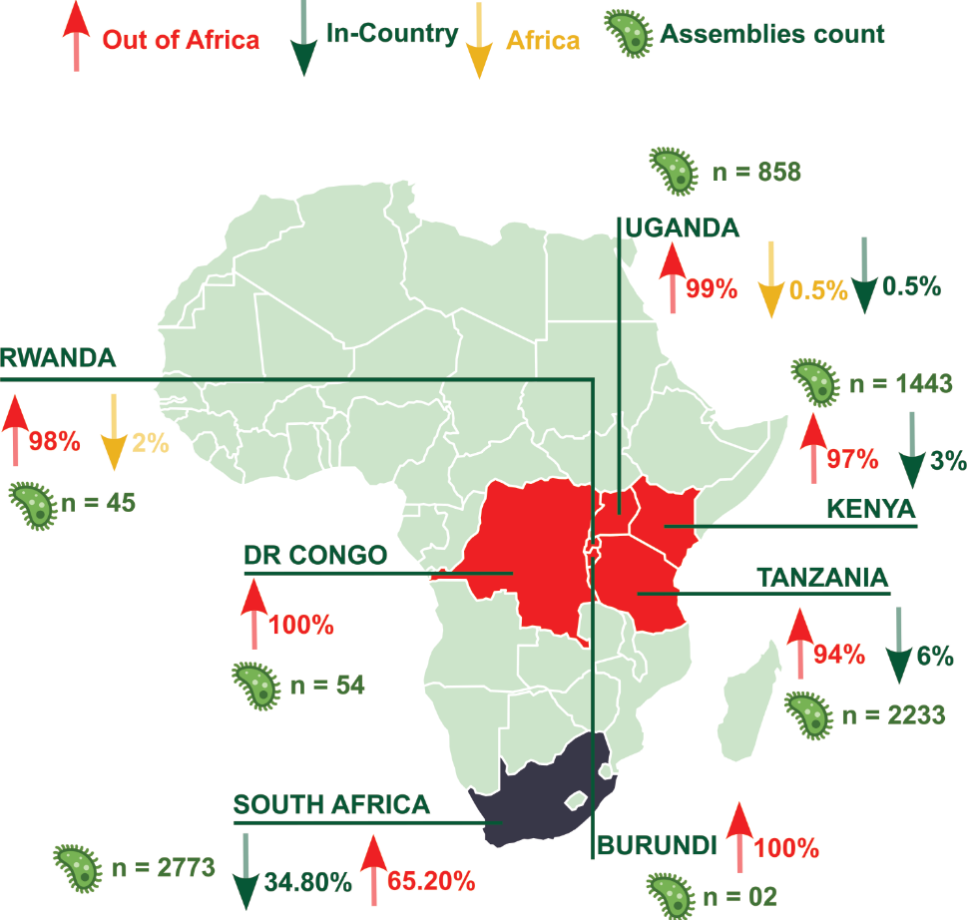
Imbalance of sequencing efforts for AMR-priority pathogens in the EAC. The map highlights a critical imbalance between isolation countries and organizations performing sequencing and analysis of AMR-GLASS priority pathogens in the East African Community (EAC) Partner States compared to South Africa, a leader on the continent in leveraging genomic and bioinformatics for public health surveillance and preparedness. Red arrow: Highlights the significant proportion of isolates sequenced by organizations outside Africa, indicating reliance on external resources. Green arrow: Indicates a smaller, but crucial, contribution from In-Country organizations to sequencing efforts. Yellow arrow: Shows the contribution from other African countries, suggesting potential regional collaboration opportunities between the EAC region and other African countries

Despite the enormous potential of pathogen NGS in addressing public health issues, EAC Partner States have yet to fully capitalize on these benefits because of various challenges. The survey assessing NPHLs’ NGS and data analysis capabilities identified challenges such as limited data storage capacity, a shortage of reagents, and a need for more trained staff in NGS data analysis, including pipelines and workflow deployment. All NPHLs express urgent needs for high-performance computing infrastructure, enhanced training programs, provision of computational resources and equipment, improved internet connectivity, and dedicated bioinformatics workspaces. These constraints extend to various facets, including under-equipped laboratory infrastructure, a scarcity of bioinformatics expertise, insufficient data management capabilities, and a pressing need for standardized protocols and stringent quality control measures [25, 26]. Another major challenge is relying heavily on external organizations and resources for regional NGS analysis. Organizations outside the EAC and Africa perform sequencing and data analysis of approximately 97% (*n* = 4,462) of the publicly available bacterial pathogen data (Fig. 5; Table 2) collected in the EAC region. This reliance on external contributors limits the EAC’s direct control over data generation, analysis, and long-term interpretation. However, for effective surveillance, it is critical to ensure that genomic data is shared and analyzed promptly. Moreover, ethical and data privacy concerns may constrain the successful implementation of NGS in public health. Overcoming these challenges is, therefore, essential for integrating systematic genomic surveillance into national infection control programs.

**Table 2:** Summary of top Organizations/Institutions sequencing AMR-GLASS pathogens sampled in the EAC

Sequencing Organization/Institution	Region	Country	Total_Genomes
Wellcome Trust Sanger Institute	Europe	United Kingdom	617
University of Wisconsin - Madison	North America	USA	499
University of South Carolina	North America	USA	483
University of Maryland	North America	USA	379
University of Queensland	Oceania	Australia	329
Washington State University	North America	USA	276
Broad Institute	North America	USA	101
Kilimanjaro Clinical Research Institute	EAC	Tanzania	76
DOE Joint Genome Institute	North America	USA	62
U.S. Army MRD–Africa	North America	USA	61
University of Bergen	Europe	Norway	38
University of Bern	Europe	Switzerland	36
University of Saskatchewan	North America	Canada	35
Massey University	Oceania	New Zealand	33
Max Planck Institute for Terrestrial Microbiology	Europe	Germany	28
University of Greifswald, Institute of Pharmacy	Europe	Germany	25
J. Craig Venter Institute (JCVI)	North America	USA	20
University of Zurich	Europe	Switzerland	20
Swiss Tropical and Public Health Institute	Europe	Switzerland	19
Los Alamos National Laboratory	North America	USA	17
Uniformed Services University of the Health Sciences	North America	USA	17
University of Copenhagen	Europe	Denmark	17
*Masinde Muliro University of Science and Technology*	EAC	Kenya	15

### Relevance of the NGS-driven public health intervention

Developing and expanding local expertise in NGS analysis is critical for several reasons. First, understanding regional pathogen diversity is crucial for effective surveillance and outbreak response. With pathogen NGS, local public health experts have valuable knowledge about the EAC region’s unique microbial diversity, allowing them to quickly identify emerging threats and implement appropriate preparedness and control measures [27]. Second, accurate and timely pathogen identification is essential for monitoring AMR patterns. By analyzing pathogen samples within the region, experts can collect critical data on the prevalence and distribution of resistance genes and inform evidence-based antimicrobial stewardship strategies [28].

### Ongoing pathogen genomics efforts in the region

Several African countries, including those within the EAC, recognize the potential of genomics and bioinformatics to transform their public health landscapes. Initiatives such as the Human Heredity and Health in Africa (H3Africa) project [29], the African Society for Laboratory Medicine (ASLM) [30], and the African Centers for Disease Control and Prevention (Africa CDC) have helped to promote research, capacity building, and technology transfer in pathogen genomics and bioinformatics in East Africa.

Within the EAC, individual Partner States have also taken strides towards promoting pathogen genomics and bioinformatics. National research institutions, universities, and health ministries are increasingly collaborating to build local expertise and infrastructure for genome sequencing and bioinformatics analysis. The EAC engages in international partnerships and consortia to pool resources, share knowledge, and harmonize efforts to address common public health challenges. Some of these initiatives are spearheaded and supported by multi-country organizations and consortia such as the Africa Pathogen Genomics Initiative (Africa PGI) [30], SeqAfrica [31], the East African WHO Regional Office, and the Centre for Epidemic Response and Innovation (CERI) [32]. They develop and sustain microbial genomics and bioinformatics capacity within the continent. Their primary focus is to strengthen AMR and infectious disease surveillance by providing necessary instrumentation, training in equipment usage, and essential bioinformatics skills for data analysis.

At the national levels, since 2017, the EAC Secretariat, in collaboration with the Bernard Nocht Institute for Tropical Medicine (BNITM) and the German Federal Ministry for Economic Cooperation and Development (BMZ) through the German Development Bank Kreditanstalt für Wiederaufbau (KfW), has been executing the EAC Regional Network of Public Health Reference Laboratories for Communicable Diseases and AMR Project across the EAC Partner States. This project includes strengthening NPHLs by facilitating the acquisition of laboratory equipment and establishing an RRMLN equipped with NGS instruments [17–19]. It also involve training national experts according to the ‘Training of Trainers’ (ToTs) concept. With this, the EAC aims to strengthen the ability of its Partner States to efficiently prepare and respond to infectious disease outbreaks, including cross-border epidemics and priority AMR pathogens.

### Strategic enhancement of the regional Bioinformatics expertise and NGS capabilities

**(i) Expanding capacity-building and training programs**.

Strategic investments must strengthen EAC’s pathogen genomic and bioinformatics capacity. Funding agencies and governmental organizations should prioritize infrastructure and training programs that foster local expertise. Training concepts tailored to the region’s needs should include hands-on practical sessions to familiarize researchers, clinicians, and laboratory technicians with NGS technologies, data analysis pipelines, and result interpretation. In addition, capacity development programs and establishing local bioinformatics centers equipped with high-performance computing infrastructure could be pivotal for a sustainable bioinformatics infrastructure. These regional genomics centers can be hubs for bioinformatics training, collaborative research, and data analysis services. They can also facilitate the development of standardized protocols and workflows for pathogen NGS analysis, ensuring the highest data quality and comparability across EAC Partner States.

**(ii) Collaborative initiatives**.

Promoting collaborative pathogen genomics research initiatives between EAC research institutions (NPHLs, academic institutions, Ministries of Health, and non-governmental organizations) and international partner organizations represents a promising avenue for addressing regional challenges in the local analysis of microbial NGS data. Such collaborations, for example, provide many benefits, such as knowledge exchange and access to shared resources unavailable in most EAC countries. They can pave the way for public health biobanks. These biobanks are critical for understanding pathogen genetic variability and susceptibility and supporting healthcare research strategies for AMR and other infectious disease surveillance, prevention, and control.

**(iii) Advocacy and policy support**.

Governments in the EAC Partner States can influence the region’s pathogen genomics and bioinformatics landscape. The EAC Partner States could collaborate to develop a regional genomic surveillance strategy. Countries, in particular, should advocate for establishing regional platforms for the transparent and timely sharing of pathogen genomic data. Such platforms should promote a culture of open data sharing, ensuring valuable information is accessible to NPHLs, health ministries, and healthcare practitioners. In addition, they should also develop legal and regulatory frameworks to ensure ethical and responsible use of pathogen genomics data. Finally, each EAC Partner State must establish a system and infrastructure to ensure harmonization in quality, performance, interpretation, and documentation of NGS data. This will facilitate and support the implementation of an in-country and sustainable pathogen genomics capacity.

## Discussion

**Fig. 6:**
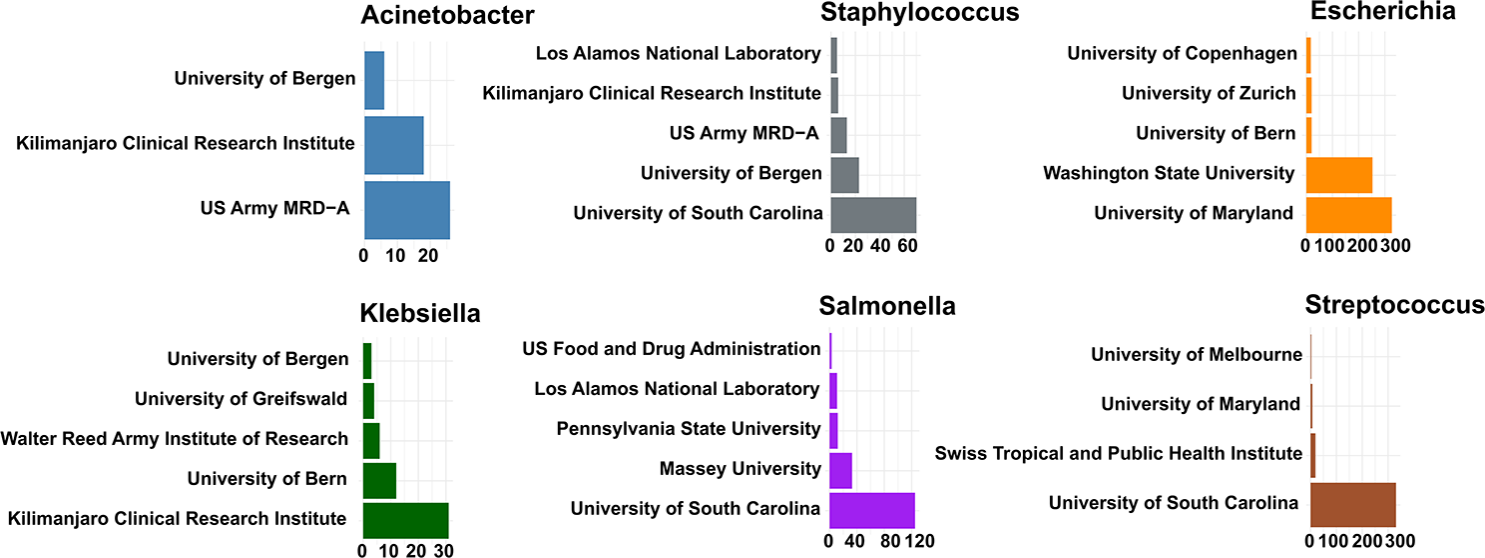
Top organizations performing NGS and submitting AMR-GLASS priority pathogens in the EAC

We have assessed and underscored the significance of pathogen NGS and bioinformatics for public health in the EAC. As highlighted in previous studies and consortium meetings [33, 34], there is a growing recognition of the need to enhance pathogen surveillance capabilities, leverage opportunities for resilient public health systems, and improve global pathogen surveillance and public health preparedness. Our study underscores the heavy dependence on external organizations and resources for sequencing and data analysis of bacterial pathogens isolated within the EAC region, as illustrated in Figs. 5 and 6. This reliance results from the shortage of local expertise and inadequate facilities and capacities for independent NGS data analysis [7]. While collaboration with international partners has been instrumental in implementing pathogen NGS and narrowing gaps with global Northern countries, building and expanding local sequencing, computing, and NGS analysis capacity is essential for a sustainable pathogen genomics and bioinformatics landscape [35]. Relying on external organizations may lead to issues with data ownership, accessibility, and delays in obtaining critical pathogen data generated outside the region. Particularly in the context of infectious disease outbreaks, prompt intervention is crucial, and any delay in sequencing and data analysis might hinder countries’ ability to make timely decisions for real-time surveillance. Therefore, establishing local data-sharing and analysis capabilities is a crucial pillar.

Despite these challenges, promising initiatives are underway in the region to pool resources, share knowledge, and harmonize efforts to address common public health challenges. High-throughput sequencing of microbial isolates has gained traction in the EAC region, partly due to global efforts during the COVID-19 pandemic [28, 34, 36]. These regional efforts and increased awareness are gradually reshaping the microbial genomics landscape in the region. However, sustained investments in local capacity-building and resource allocation are needed to achieve NGS self-sufficiency and fully utilize the potential of pathogen genomics to guide effective public health interventions, outbreak preparedness and response. Countries that have consistently invested in NGS technology and laboratory infrastructure have demonstrated rapid national responses to infectious disease threats, as evidenced by their response to the COVID-19 pandemic [8, 10]. A notable example is South Africa, which concentrates nearly 40% of the continent’s genomics capacities while demonstrating high pathogen sequencing capabilities [25].

We compared the EAC pathogen data with data from South Africa, a pioneer on the continent with robust pathogen genomic infrastructure and success stories of leveraging these genomic capacities to improve public surveillance and preparedness. This data shows clearly that, due to the deployment of local pathogen genomic and bioinformatics capacity, the in-country-sequencing and data analysis capabilities are substantially better (~ 35%) in South Africa than the average (~ 3%) in the EAC region, supporting that the lack of local skill personal and infrastructure is associated with low in-country sample analysis in these countries (Fig. 6). This reinforces the importance of strategic investment in genomic infrastructure, particularly in regions with significant healthcare burdens. South Africa’s robust genomic capacity positions it as a leader in pathogen surveillance and outbreak response within Africa and highlights the potential for leveraging such capabilities to address broader global health challenges. Therefore, pathogen genomics and data analysis, such as bioinformatics, need to be mainstreamed locally to benefit communities effectively. Systematically shipping samples abroad neither supports the regional public health systems nor benefits the host institutions on the continent. Addressing these gaps is crucial by prioritizing sustained regional capacity-building efforts and funding initiatives within Africa and the EAC.

## Conclusion

In conclusion, assessing pathogen genomic capabilities in the EAC highlights critical challenges and underscores the urgent need for strategic interventions to improve public health surveillance, intervention, and preparedness. Addressing these challenges requires concerted efforts and a multi-level strategy, including collaborative initiatives involving local universities, research institutions, and international partners. Strengthening local NGS capacity and expertise, establishing regional data-sharing and analysis platforms, and developing regulatory frameworks for pathogen data management are essential steps. Enhanced regional collaboration in setting quality standards for processing, reporting, and interpreting pathogen genomics data can optimize these initiatives. These efforts will empower NPHLs and national laboratories to independently perform pathogen sequencing and data analysis, improving disease surveillance, outbreak preparedness, and response capabilities. This will ultimately support the development of sustainable NGS data-driven national strategic plans to strengthen public health systems in the EAC Partner States.

### Electronic supplementary material

Below is the link to the electronic supplementary material.

Supplementary Material 1

Supplementary Material 2

Supplementary Material 3

## Data Availability

No datasets were generated or analysed during the current study.
