# Supplementary material for: Assessment of the pathogen genomics landscape highlights disparities and challenges for effective AMR Surveillance and outbreak response in the East African community
Source: BMC Public Health. 2024 Jun 5;24:1500. doi: 10.1186/s12889-024-18990-0 (PMC11151545; doi:10.1186/s12889-024-18990-0)
Supplement: Supplementary file 1 — Supplementary Material 1 [file 12889_2024_18990_MOESM1_ESM.pdf]

In order to submit this form, you should open it with Adobe Acrobat Reader.

SURVEY TO ASSESS Next-Generation Sequencing (NGS) Capacity of the National Public Health Laboratories (NPHLs) in the East African Community (EAC)

1. NPHLs Informations

1.1. EAC Partner State

Please Select

1.2. Name of the National Public Health Laboratory

1.3. Phone Number

Country Code

Phone Number

1.4. E-Mail *

example@example.com

1.5. Contact/Focal Person

First Name

Last Name

2. NGS Equipments in the NPHLs

Which of the following NGS instruments are available in your laboratory?

2.1. Illumina NGS Platforms *

- ☐ iSeq100
- ☐ MiniSeq

Create your own automated PDFs with Jotform PDF Editor- It's free

- ☐ MiSeq
- ☐ NextSeq 550
- ☐ NextSeq 1000/2000
- ☐ NovaSeq 6000
- ☐ NovaSeq X
- ☐ HiSeq
- ☐ None of the above

2.2. Oxford Nanopore Technologies (ONT) NGS Platforms *

- ☐ MinION
- ☐ PromethION
- ☐ SmidgION (Smartphone Sequencer)
- ☐ GridION
- ☐ None of the above

2.3. Pacific Biosciences (PacBio) NGS Platforms *

- ☐ Sequel system
- ☐ Sequel II system
- ☐ Sequel IIe system
- ☐ Revio system
- ☐ Onso system
- ☐ None of the above

3. Bacterial Samples Processing Capabilities

3.1. Which of the following can be performed by your Lab? *

- ☐ Bacterial genomic DNA isolation
- ☐ Library preparation
- ☐ Bacterial whole genome sequencing
- ☐ Bacterial genome assembly and annotation
- ☐ Antimicrobial resistance (AMR) genotyping
- ☐ Data submission (e.g. raw reads, genome sequences, ...) in public repositories, including GenBank, ENA, SRA.

3.2. How many of these pathogens isolates have been sequenced yearly in your lab?

	2019	2020	2021	2022	2023
**Acinetobacter spp.**					
**Escherichia coli**					

Create your own automated PDFs with Jotform PDF Editor- It's free
Jotform

Klebsiella pneumoniae					
Neisseria gonorrhoeae					
Salmonella spp.					
Shigella spp.					
Staphylococcus aureus					
Streptococcus pneumoniae					
Other non-GLASS-priority pathogens					

3.3. How many of these pathogens isolates have been analyzed yearly in your lab?

	2019	2020	2021	2022	2023
Acinetobacter spp.					
Escherichia coli i					
Klebsiella pneumoniae					
Neisseria gonorrhoeae					
Salmonella spp.					
Shigella spp.					
Staphylococcus aureus					
Streptococcus pneumoniae					
Other non-GLASS-priority pathogens					

4. Bioinformatics Computing Capacity Assessment

4.1. Do you have a dedicated bioinformatics computing facility or workspace in the laboratory? *

○ Yes ○ No

4.2. What computational resources are available in your laboratory? (Check all that apply)

☐ High-performance computing (HPC) cluster
☐ Standalone servers
☐ Workstations

Create your own automated PDFs with Jotform PDF Editor- It's free

☐ Cloud computing services
☐ None

4.3. Do you have access to a reliable internet connection for data transfer and online analysis? *

○ Yes ○ No

4.4. Do you have staff with bioinformatics expertise and training in your laboratory? *

○ Yes ○ No

4.5. Do you have access to a reliable internet connection for data transfer and online analysis? *

○ Yes ○ No

4.6. If yes, please rate the level of bioinformatics expertise among your staff:

☐ Novice (Limited or no experience)
☐ Intermediate (Basic skills and experience)
☐ Advanced (Proficient with substantial experience)
☐ Expert (Highly skilled with extensive experience)

4.7. Please specify the roles of staff with bioinformatics expertise (check all that apply):

☐ NGS Data analysis
☐ Sequence analysis
☐ Pipelines/Workflows development
☐ Database management
☐ Training and capacity building

4.8. Are there any collaborations with external organizations or institutions for bioinformatics support? *

○ Yes ○ No

You are almost done...

Create your own automated PDFs with Jotform PDF Editor- It's free
Jotform
